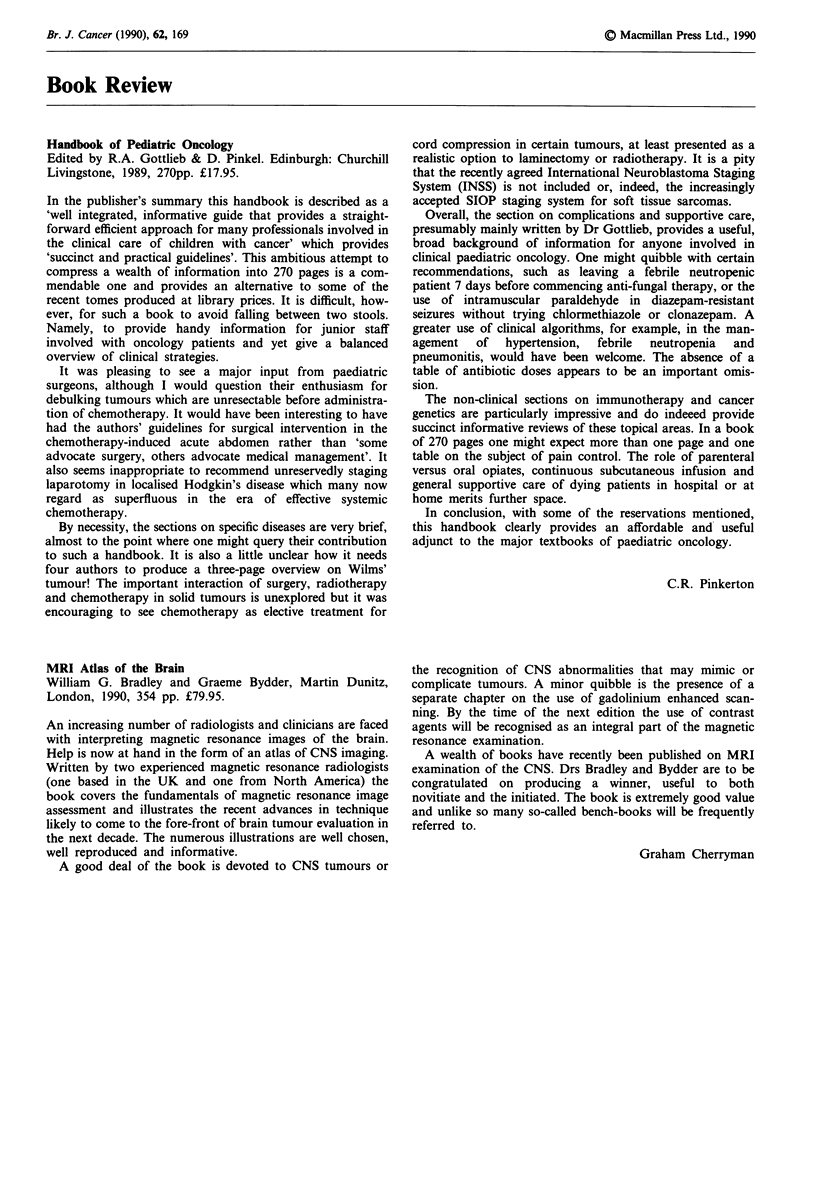# Handbook of Peadiatric Oncology

**Published:** 1990-07

**Authors:** C.R. Pinkerton


 © Macmillan Press Ltd., 1990

### Book Review

**Handbook of Pediatric Oncology**
Edited by R.A. Gottlieb & D. Pinkel. Edinburgh: Churchill Livingstone, 1989, 270pp. £17.95.

In the publisher's summary this handbook is described as a 'well integrated, informative guide that provides a straightforward efficient approach for many professionals involved in the clinical care of children with cancer' which provides 'succinct and practical guidelines'. This ambitious attempt to compress a wealth of information into 270 pages is a commendable one and provides an alternative to some of the recent tomes produced at library prices. It is difficult, however, for such a book to avoid falling between two stools. Namely, to provide handy information for junior staff involved with oncology patients and yet give a balanced overview of clinical strategies.

It was pleasing to see a major input from paediatric surgeons, although I would question their enthusiasm for debulking tumours which are unresectable before administration of chemotherapy. It would have been interesting to have had the authors' guidelines for surgical intervention in the chemotherapy-induced acute abdomen rather than 'some advocate surgery, others advocate medical management'. It also seems inappropriate to recommend unreservedly staging laparotomy in localised Hodgkin's disease which many now regard as superfluous in the era of effective systemic chemotherapy.

By necessity, the sections on specific diseases are very brief, almost to the point where one might query their contribution to such a handbook. It is also a little unclear how it needs four authors to produce a three-page overview on Wilms' tumour! The important interaction of surgery, radiotherapy and chemotherapy in solid tumours is unexplored but it was encouraging to see chemotherapy as elective treatment for cord compression in certain tumours, at least presented as a realistic option to laminectomy or radiotherapy. It is a pity that the recently agreed International Neuroblastoma Staging System (INSS) is not included or, indeed, the increasingly accepted SIOP staging system for soft tissue sarcomas.

Overall, the section on complications and supportive care, presumably mainly written by Dr Gottlieb, provides a useful, broad background of information for anyone involved in clinical paediatric oncology. One might quibble with certain recommendations, such as leaving a febrile neutropenic patient 7 days before commencing anti-fungal therapy, or the use of intramuscular paraldehyde in diazepam-resistant seizures without trying chlormethiazole or clonazepam. A greater use of clinical algorithms, for example, in the management of hypertension, febrile neutropenia and pneumonitis, would have been welcome. The absence of a table of antibiotic doses appears to be an important omission.

The non-clinical sections on immunotherapy and cancer genetics are particularly impressive and do indeeed provide succinct informative reviews of these topical areas. In a book of 270 pages one might expect more than one page and one table on the subject of pain control. The role of parenteral versus oral opiates, continuous subcutaneous infusion and general supportive care of dying patients in hospital or at home merits further space.

In conclusion, with some of the reservations mentioned, this handbook clearly provides an affordable and useful adjunct to the major textbooks of paediatric oncology.

C.R. Pinkerton